# Development and evaluation of a health‐related quality‐of‐life tool for dogs with Cushing's syndrome

**DOI:** 10.1111/jvim.15639

**Published:** 2019-10-29

**Authors:** Imogen Schofield, Dan G. O'Neill, Dave C. Brodbelt, David B. Church, Rebecca F. Geddes, Stijn J. M. Niessen

**Affiliations:** ^1^ Pathobiology and Population Science The Royal Veterinary College Hatfield United Kingdom; ^2^ Clinical Sciences and Services The Royal Veterinary College Hatfield United Kingdom; ^3^ The VetCT Telemedicine Hospital, St John's Innovation Centre Cambridge United Kingdom

**Keywords:** canine, Cushing's disease, endocrinology, trilostane, welfare

## Abstract

**Background:**

Clinical signs and consequences of Cushing's syndrome are likely to impact upon a dog's life. Quantification of this impact on a dog's health‐related quality‐of‐life (HRQoL) could contribute to optimized disease management.

**Hypothesis/objectives:**

To develop a novel HRQoL tool to aid assessment of dogs with Cushing's syndrome and to evaluate factors that impact upon dogs living with this disease.

**Animals:**

Two hundred and ten dogs with Cushing's syndrome and 617 dogs without Cushing's syndrome.

**Methods:**

Cross‐sectional study design. Dog owners answered questions relating to the HRQoL of their dogs which were refined to develop the final tool. The tool was analyzed for reliability, validity, and interpretability, including Cronbach's alpha and principal components analysis. Factors impacting upon the HRQoL of dogs with Cushing's syndrome were assessed using appropriate nonparametric tests.

**Results:**

The tool was refined from 32 questions to 19 and showed good internal consistency (α = .83). Owners rated questions related to “owner impact” as more important and those related to demeanor as less important. There was a positive correlation between the tool score of dogs with Cushing's syndrome and owner's assessment of their dog's quality‐of‐life (*r* = .41, *P* < .001). Dogs currently on treatment with trilostane had a statistically better HRQoL (.33, interquartile range [IQR] .23–.44) than those not receiving trilostane (.36, IQR .33–.54, *P* = .04).

**Conclusions and Clinical Importance:**

The developed tool quantifies the HRQoL of dogs with Cushing's syndrome and could assist clinicians in the clinical assessment of dogs with Cushing's syndrome.

**Abbreviations**
HRQoL: health‐related quality‐of‐life
ICC: intraclass correlation coefficient
IQR: interquartile range
KMO: Kaiser–Meyer–Olkin measure of sampling adequacy
PCA: principal components analysis

## 1 INTRODUCTION

Assessing the quality‐of‐life of animals is an integral role of a veterinarian and is required during decision‐making on treatment and euthanasia to optimize the health and welfare of animals under their care.^1^ In the current study, health‐related quality‐of‐life (HRQoL) refers to the state of an individual animal's life as thought to be perceived by them at a point in time by their owner. This includes the physical, social, and environmental needs and impacts which are reflected by the animal's health and behavior.^2^ A fundamental issue currently is that assessment of welfare or quality‐of‐life is not standardized or validated. The assessment is nearly always subjectively and compassionately inferred by veterinary professionals and animal owners. Consequently, quantification is increasingly promoted to optimize and standardize decision‐making in this area.^2^, ^3^, ^4^ The British Veterinary Association's Animal Welfare Strategy highlighted the use of welfare assessments as 1 of their 6 priorities, which includes the use of practice‐based quality‐of‐life assessments.^5^ In human medicine, formal HRQoL measures are commonly implemented in practice to provide additional information about the dogs without solely assessing laboratory results or clinical outcomes.^6^ It is accepted that lack of assessment on a dog's HRQoL could result in inadequate relief from suffering and suboptimal clinical decision‐making^4^, ^7^ with an awareness that the severity of the clinical signs affecting an individual might correlate poorly with results of routine blood tests.^8^ Practice‐based quality‐of‐life tools developed for veterinary medicine have followed the methodology produced in human medicine to measure HRQoL.^2^, ^9^, ^10^, ^11^, ^12^, ^13^, ^14^

Cushing's syndrome in dogs results from excessive circulating glucocorticoids. The disease is clinically characterized variably by polyuria and polydipsia, polyphagia, bilateral alopecia, muscle atrophy with generalized weakness, hepatomegaly, systemic hypertension, and lethargy.^15^, ^16^ These clinical signs can all impact upon dogs' as well as to their owner's lives. Currently no tool to quantify the impact or the long‐term residual effects of Cushing's syndrome on a dog's life has been published. Therefore, such a tool is warranted to optimize disease management, taking the financial and emotional strain of Cushing's syndrome to the owner into consideration in its design. Any negative impact of disease and treatment on the owner could lead to cessation of treatment or even euthanasia.

The aims of this study were to develop a novel HRQoL tool to aid clinical assessment of dogs with Cushing's syndrome and to evaluate factors that might impact upon the quality‐of‐life of dogs with this disease. It was hypothesized that Cushing's syndrome cases not receiving treatment would have a poorer HRQoL.

## 2 METHODS

HRQoL tool development followed a standard psychometric process of item identification, selection, and refinement.^9^, ^10^, ^17^, ^18^ An item was defined as any aspect of Cushing's syndrome and its management that could potentially impact on a dog's HRQoL. Health‐related quality‐of‐life tools must be shown to be valid, reliable, and interpretable before recommending their use in a clinical context.^8^, ^12^, ^18^ Ethical approval was granted by the Royal Veterinary College Ethics and Welfare Committee (URN 2015 1373).

### 2.1 CushQoL‐pet development

#### 2.1.1 Item identification

Items potentially impacting the HRQoL of Cushing's syndrome in dogs were identified through a variety of sources. A focus group discussion, face‐to‐face interviews, and telephone interviews were conducted with veterinarians (16 primary‐care practitioners, 2 internal medicine specialists, and a dermatologist), 2 veterinary nurses and 13 owners of dogs with Cushing's syndrome. A list of guiding, open‐ended questions regarding possible effects of this disease on HRQoL was applied, with all the answers transcribed for qualitative interpretation. A broader overview of potential items was identified from review of the relevant literature, an interview with a human Cushing's syndrome patient and 2 developers of a human Cushing's syndrome HRQoL tool^17^ as well as review of 20 randomly selected electronic health records from primary‐care caseloads of dogs with Cushing's syndrome.^19^

#### 2.1.2 Item selection

A questionnaire was designed to explore all identified items. A pilot of the questionnaire was performed by owners of dogs both with and without Cushing's syndrome, veterinarians, specialists in animal behavior and welfare, and veterinary epidemiologists to identify ambiguous, unnecessary, or missing questions that needed revision. Questions were designed from the transcripts to reflect the phrases and words used by owners and veterinarians.

The final amended questionnaire was uploaded to an online survey tool (SurveyMonkey, San Mateo, California). Owners of dogs, both with and without Cushing's syndrome, were eligible to complete the questionnaire. Responses were excluded if they were incomplete or had been completed retrospectively by the owner regarding deceased animals. The questionnaire was promoted via veterinary practice client e‐mails and practice posters, website links, and social media posts. The Royal Veterinary College, Veterinary Information Network, Dechra Veterinary Products Ltd, Vets4Pets, Independent Vet Care, Dogs Trust, and the Dog Science Group all promoted the questionnaire.

Owners were asked to describe the frequency of each specified item impacting on their dog's life over the previous week. Responses were assigned a score (all the time (3), often (2), occasionally (1), never (0) for negatively phrased questions). The scores were reversed in positively phrased questions.

#### 2.1.3 Item refinement

To develop the finalized “CushQoL‐pet” tool, the questions included were refined based on statistical analysis of the responses.

1. Chi‐squared analysis was performed on each item, comparing the results from dogs with and without Cushing's syndrome. Items with at least weak evidence of differences between the 2 groups were retained as these were deemed specific to the impact of Cushing's syndrome on HRQoL (*P* < .20).
2. Internal consistency of the questionnaire was measured by Cronbach's alpha, using only the responses of owners of dogs with Cushing's syndrome. Internal consistency indicates the reliability of the questions to measure the same latent concept.^20^ In the context of this study, the latent concept was “HRQoL.” Cronbach's alpha was calculated using a 1‐way repeated measure analysis of variance model, with HRQoL question responses functioning as the repeated measure. Initially, correlations were examined in an inter‐item correlation matrix to assess how much each individual question responses correlated with all included questions. Low correlations (*r* < .30) were deemed poor and those questions were removed if the overall Cronbach's alpha coefficient increased after removal. Correlations between pairs of questions were examined to check whether they were deemed highly correlated (*r* > .60), suggesting the same information is being captured twice therefore falsely raising the internal consistency of the tool.^21^ The question with the smallest effect on the Cronbach's alpha was removed. An overall test Cronbach's alpha of α > .70 for the retained questions was deemed an appropriate internal consistency.^22^

Internal validation using the dog's name, age, sex, and breed prevented duplication of responses relating to a single dog. If duplicates were found, the earliest response was used for analysis.

### 2.2 Interpretation, validation, and reliability of CushQoL‐pet

After refinement of the tool, the finalized questions were utilized to produce a combined score of HRQoL, rating between 0 and 1 (0 indicating the best possible HRQoL and 1 indicating the worst possible). The scoring was calculated as follows:

CushQoL−petScore=∑of the question scores/total maximum score

Questions were included in the questionnaire to assess the validity of the tool. One question asked owners to describe their dog's current quality‐of‐life on a 7 point scale (from “as good as it could be” to “as poor as it could possibly be”), to assess construct validity. Correlations were analyzed with Spearman's rank correlation. Wilcoxon rank‐sum and Kruskal‐Wallis tests compared CushQoL‐pet scores of dogs with and without Cushing's syndrome. Dogs with and without Cushing's syndrome were further categorized by (1) age group (<7, 7–11, >11 years), and (2) health status (“healthy” and “not‐healthy” group, if a disease other than Cushing's syndrome was reported by the owner).

Principal components analysis (PCA) assessed the underlying structure and identified subsets within the CushQoL‐pet tool. The principal components describing the largest amount of data variation were retained for further interpretation. Retention was based on visualization of the decreasing proportion of data variance described by each principal component, using a scree‐plot.^21^, ^23^ For each retained principal component, the HRQoL question loading scores were analyzed and interpreted to observe those with the greatest influence on each principal component. Loadings closest to −1 or 1 for an item indicate a strong influence on the component.^21^ A loading of ≥0.3 was selected as an appropriate cutoff.^10^, ^24^ The internal consistency of the identified subsets was examined with Cronbach's alpha. The Kaiser–Meyer–Olkin (KMO) measure of sampling adequacy was used postestimation to assess whether the patterns of correlation from the PCA were relatively compact and therefore the results were reliable.^25^ KMO values >.50 indicate adequate sampling.

To assess the reliability of the tool, inter‐rater measures of the questionnaire were assessed on 13 dogs, with pairs of owners of the same dog completing the questionnaire independently of each other. Intra‐rater reliability was carried out with 15 owners to examine the stability of the responses from the same person carrying out the questionnaire at an interval of 2 weeks, with no changes to the management of their dog's Cushing's syndrome. Paired scores were assessed with the intraclass correlation coefficient (ICC), with results interpreted as poor reliability (<.50), moderate (.50 to <.75), good (.75 to <.90), and excellent (≥.90).^26^ Bland–Altman plots were analyzed to assess score agreement between 2 owners and repeat response at a 2 week interval.^27^, ^28^ These scores helped inform the suitability of questions for inclusion in the tool. Seventy‐one owners of dogs with Cushing's syndrome repeated the CushQoL‐pet at least 3 months after their first response. These follow‐up responses were used to assess the test‐retest reliability of the score. Owners answered additional questions relating to changes in their dog's management and quality‐of‐life since their previous response. Correlation between the differences in the 2 CushQoL‐pet scores and the owner assessment of a change in quality‐of‐life were assessed with Spearman's rank correlation.

### 2.3 Evaluation of factors impacting the HRQoL of dogs with Cushing's syndrome

The online questionnaire also asked owners to provide some additional information as well as the core questions. Owners assessed the importance of each HRQoL question to themselves and their dog (very important (4), important (3), moderately important (2), low importance (1), not at all important (0)).^10^ Inter‐rater and intra‐rater reliability assessments of owner reported importance were also carried out as described above. Additional demographic information relating to their dog included age, breed, sex, weight, insurance status, and other health concerns. Specific questions about owners included owner lifestyle, time spent with their dog, and whether they were the primary care‐giver. Owners of dogs with Cushing's syndrome were asked disease‐specific questions, including treatment currently received, time since diagnosis and how their dog's quality‐of‐life had changed since their diagnosis. Differences between dogs with and without Cushing's syndrome were analyzed using chi‐squared analysis. Factors impacting upon the CushQoL‐pet score were assessed using nonparametric analyses (either Wilcoxon rank‐sum test or Krukshal‐Wallis test). Statistical significance was set at <.05.

## 3 RESULTS

### 3.1 CushQoL‐pet development

#### 3.1.1 Item identification

From the focus group discussions, interviews, and reviews of relevant literature, 32 HRQoL items specific to dogs with Cushing's syndrome were identified.

#### 3.1.2 Item selection

A questionnaire was developed incorporating the 32 items identified. During pretesting, 6 questions deemed inappropriate or ambiguous were removed and 3 were reworded for clarification resulting in 26 HRQoL questions included in the online questionnaire.

#### 3.1.3 Item refinement

Owners of dogs with (n = 237) and without Cushing's syndrome (n = 699) completed the online questionnaire. There were 95 incomplete responses that were excluded from analysis: 13 (6.2%) with Cushing's syndrome and 82 (11.7%) without. Eight owners of dogs with Cushing's syndrome answered the study about dogs that were no longer alive and 6 duplicate responses were identified and were removed, resulting in 210 responses related to dogs with Cushing's syndrome and 617 for dogs without Cushing's syndrome. No owners identified their dog as having iatrogenic Cushing's.

When comparing responses to the HRQoL questions by owners of dogs with or without Cushing's syndrome, no difference was observed in the responses to “medication stress” or “off food” so these were removed from the tool (*P* = .22 and *P* = .33, respectively). Based on correlations between an individual question and all other HRQoL questions, 3 questions were removed (“frequency of urination”, “vet stress,” and “begs for food”) as they were poorly correlated to the other items (*r* = .29, .23, and .22, respectively), improving the internal consistency of the score. A number of HRQoL questions were found to be highly correlated with each other: “thirsty” with “emptying water bowl” (*r* = .68) and “weak” with “struggles to walk” (*r* = .63). “Emptying water bowl” and “weak” were removed from the score as these resulted in the least change in Cronbach's alpha coefficient. The process of item refinement reduced the number of items from 26 to 19 in the final CushQoL‐pet tool, with a Cronbach's alpha of α = .83 (Table 1).

**Table 1:** Final items included in the Cushing's syndrome HRQoL tool (CushQoL‐pet) after question refinement

1 My dog is excessively thirsty All the time (3), often (2), occasionally (1), never (0)
2 My dog urinates in the house All the time (3), often (2), occasionally (1), never (0)
3 My dog is excessively hungry All the time (3), often (2), occasionally (1), never (0)
4 My dog pants excessively All the time (3), often (2), occasionally (1), never (0)
5 My dog appears to be gaining weight All the time (3), often (2), occasionally (1), never (0)
6 My dog is depressed and quiet All the time (3), often (2), occasionally (1), never (0)
7 My dog has no energy All the time (3), often (2), occasionally (1), never (0)
8 My dog doesn't want to interact with other people / dogs All the time (3), often (2), occasionally (1), never (0)
9 My dog is reluctant to play with me All the time (3), often (2), occasionally (1), never (0)
10 My dog seems disorientated/confused All the time (3), often (2), occasionally (1), never (0)
11 My dog's hair coat is in a poor condition All the time (3), often (2), occasionally (1), never (0)
12 My dog's skin appears to be uncomfortable (eg, dry/tight) All the time (3), often (2), occasionally (1), never (0)
13 My dog appears to be in poor physical condition (eg, muscle loss/big belly) All the time (3), often (2), occasionally (1), never (0)
14 I feel my dog's appearance gets negative comments All the time (3), often (2), occasionally (1), never (0)
15 My dog struggles to walk very far All the time (3), often (2), occasionally (1), never (0)
16 I worry about the future health of my dog All the time (3), often (2), occasionally (1), never (0)
17 Mine and my dog's daily routine is being disrupted All the time (3), often (2), occasionally (1), never (0)
18 I feel I am struggling to manage my dog's health All the time (3), often (2), occasionally (1), never (0)
19 Currently I feel there is a strong bond between me and my dog All the time (0), often (1), occasionally (2), never (3)

All 32 items initially identified for inclusion in the online questionnaire which were subsequently retained or excluded from the final tool, CushQoL‐pet, are outlined (Supporting Information).

### 3.2 Interpretation, validation, and reliability of CushQoL‐pet

**Figure 1:**
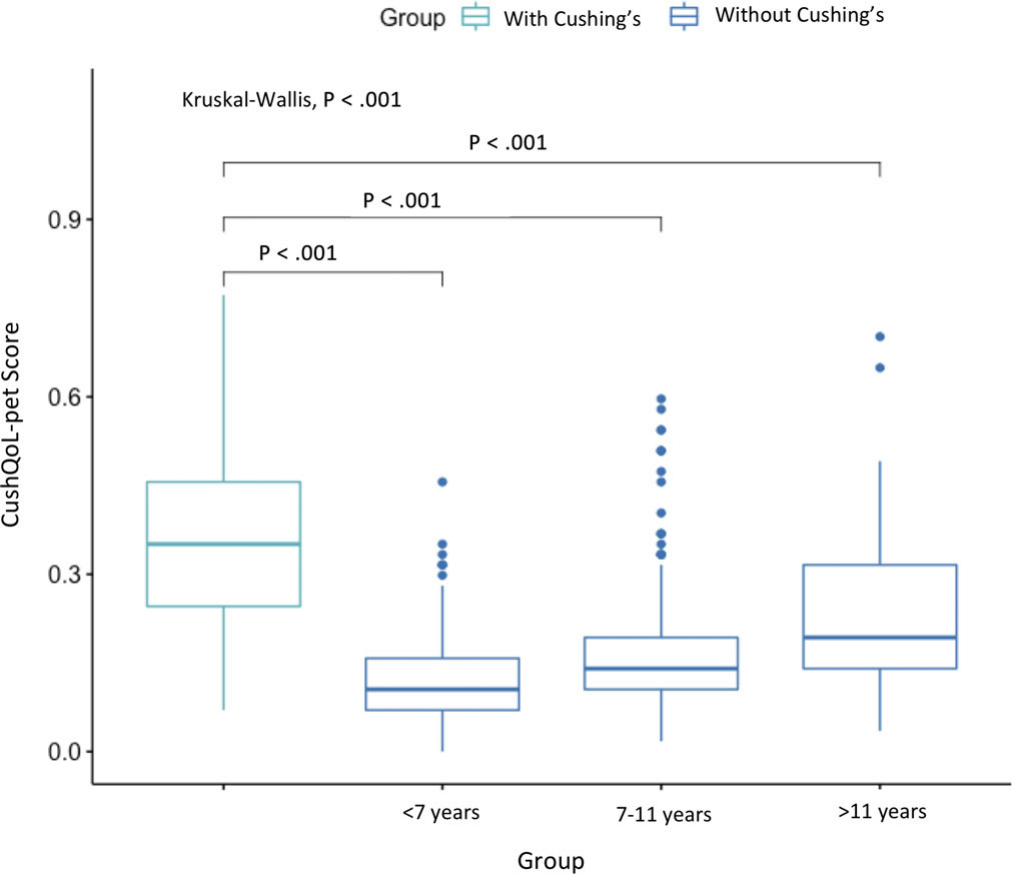
CushQoL‐pet scores in dogs with Cushing's syndrome (median .35, IQR .25–.46) and those without Cushing's syndrome, separated by age groups (<7 years [.11, IQR .07–.16]; 7–11 years [.14, IQR .11–.19]; >11 years [.19, IQR .14–.32])

The median HRQoL score for dogs with Cushing's syndrome using the final tool was .35 (range .07–.77, interquartile range [IQR] .25–.46). Dogs without Cushing's syndrome had a median score of .12 (range .00–.70, IQR .09–.19, *P* < .001). For dogs with Cushing's syndrome, no difference in the HRQoL tool score was found between age groups (*P* = .84). Increasing HRQoL scores of dogs with Cushing's syndrome were seen with increasing owner assessment scores (Spearman's rho = .40, *P* < .001). Health‐related quality‐of‐life scores among the 3 non‐Cushing's syndrome age groups and dogs with Cushing's syndrome were statistically different (*P* < .001) (Figure 1).

Principal components analysis was conducted on the final 19 questions to identify grouping of questions within the tool and highlighted 3 principal components accounting for 58.2% of the data which were retained for further analysis. Items clustering on differing components suggested that component 1 represents the dog's demeanor (depressed [Q6], no energy [Q7], and reluctance to play [Q9]; Cronbach's α = .79), component 2 the dog's clinical signs of Cushing's syndrome (thirst [Q1], urination [Q2], and hunger [Q3]; Cronbach's α = .66), and component 3 the dog's appearance (hair coat [Q11], skin [Q12], and poor physical condition [Q13]); Cronbach's α = .71 (Table 2). Kaiser–Meyer–Olkin postestimation was .82 indicating adequate sample size and reliable results.

**Table 2:** Principal component analysis factor loadings to the 19 questions of the CushQoL‐pet. Principal component (PC) 1, PC2, and PC3 explained 58.2% of the data variance. Question loadings closest to −1 or 1 are highlighted, indicate the strongest influence on that principal component

CushQoL‐pet question	PC1	PC2	PC3
Thirsty	.13	.39	.18
Urinates in the house	.11	−.19	.16
Hungry	.11	.35	.13
Pants	.13	.37	.29
Weight gain	.16	.38	.30
Depressed	.33	−.22	−.01
No energy	.35	−.11	.11
Does not interact	.30	−.26	.18
Reluctant to play	.38	−.29	.04
Disorientated	.25	−.26	.04
Poor hair coat	.20	.20	−.42
Dry/tight skin	.19	.26	−.42
Poor physical condition	.21	.10	−.10
Negative comments	.23	.20	−.39
Struggles to walk	.32	−.07	.12
Future health concern	.18	.16	.11
Disrupted routine	.29	.02	.04
Owner struggling	.29	−.01	−.02
Dog‐owner bond	−.15	.23	.19

**Figure 2:**
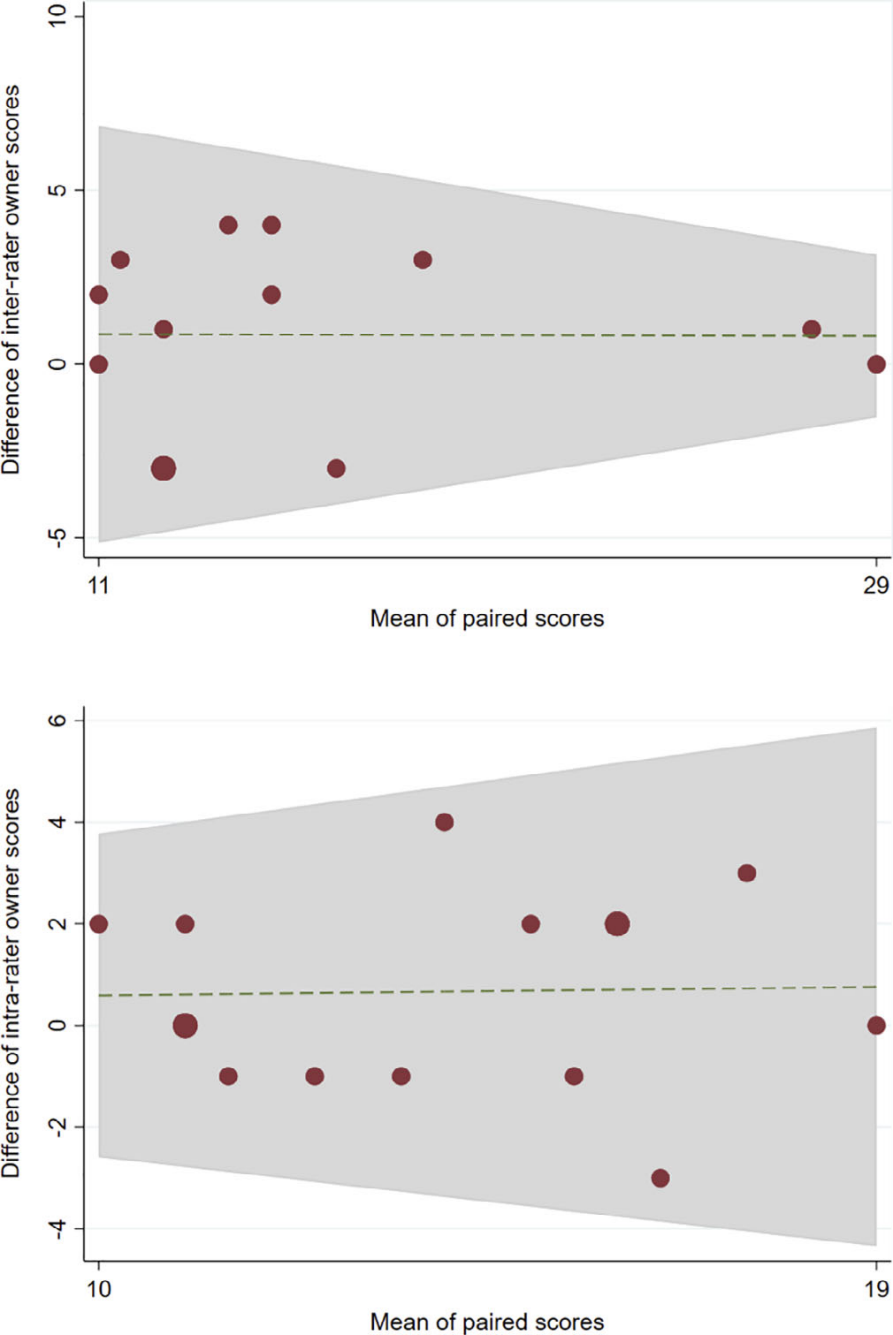
Bland‐Altman plots of inter‐rater (n = 13) and intra‐rater (n = 15) owner scores of CushQoL‐pet

When assessing the reliability of the HRQoL questions, inter‐rater (n = 13, ICC = .88, 95% CI .55–.97) and intra‐rater agreement (n = 15, ICC = .78, 95% CI .49–.92) indicated good reliability. There was also a correlation with Bland‐Altman plots, which suggest good agreement of paired owner and repeated owner responses for the HRQoL questions (Figure 2). Test‐retest results showed a correlation between the difference in the 2 CushQoL‐pet scores (Spearman's rho = .64, *P* < .001) and how owners described a change in their dogs quality‐of‐life (Table 3). Test‐retest results also showed a significant correlation between owners assessment of quality‐of‐life and the CushQoL‐pet score (rho = .69, *P* < .001).

**Table 3:** Median test‐retest CushQoL‐pet scores at initial completion and at 3 month follow‐up, stratified by owner assessment of change in their dog's quality‐of‐life in this time period (n = 71). Spearman's rho = .64, *p* < .001

Owner assessment (n = 71)	Initial CushQoL‐pet score (median, IQR)	3 month follow‐up CushQoL‐pet score (median, IQR)	Score difference (median)
A great deal better (n = 7)	.35 (.28–.42)	.18 (.11–.25)	−.16
Quite a lot better (n = 11)	.37 (.16–.54)	.21 (.07–.39)	−.14
A little better (n = 16)	.34 (.26–.44)	.27 (.19–.37)	−.08
No difference (n = 15)	.32 (.21–.42)	.28 (.18–.42)	+.02
A little worse (n = 14)	.39 (.23–.42)	.41 (.28–.46)	+.04
Quite a lot worse (n = 2)	.51 (.47–.54)	.54 (.52–.56)	+.04
A great deal worse (=6)	.31 (.25–.42)	.40 (.39–.70)	+.08

### 3.3 Evaluation of HRQoL in dogs with Cushing's syndrome

Overall respondents originated from the United Kingdom (n = 622, 69.9%), United States (184, 20.7%), and 25 other countries (84, 9.4%). Median age of dogs with Cushing's syndrome was 11 years (IQR 9–13) and dogs without Cushing's syndrome was 7 years (IQR 4–10). The most represented breeds of dog with Cushing's syndrome were crossbreeds (n = 37, 17.6%), Border Terriers (14, 6.7%), Bichon Frise (12, 5.7%), and Jack Russell Terriers (9, 4.3%). Dogs with Cushing's syndrome were less likely to be insured (n = 65, 31.1%) than those without Cushing's syndrome (388, 57.0%, *P* = .002). Owners of dogs with Cushing's reported to spend >8 hours a day with their dogs (145, (69.1%), with 206 (98.1%) describing themselves as the primary‐care giver to their dog. Owners of dogs with and without Cushing's syndrome differed in how they viewed their dogs current quality‐of‐life (*P* < .001), with the reported quality‐of‐life for dogs with Cushing's syndrome generally poorer. Eighty (38.1%) owners of dogs with Cushing's syndrome described their dog's current quality‐of‐life “as good as it could possibly be.” Most dogs with Cushing's syndrome were first diagnosed over 12 months previous to the questionnaire (104, 49.5%), with 160 (76.2%) currently on trilostane (Vetoryl Capsules, Dechra Veterinary Products Ltd, Shrewsbury, United Kingdom) treatment with 110 (68.8%) receiving their trilostane once daily. When owners were asked about their dog developing an Addisonian crisis, 43 (20.5%) did not know what an Addisonian crisis was and 52 (24.8%) never worried about it. Owners of 165 (80.5%) dogs with Cushing's syndrome felt they understood the disease either “very well” or “fairly well.” The average time to complete the full online questionnaire was 6 minutes.

**Figure 3:**
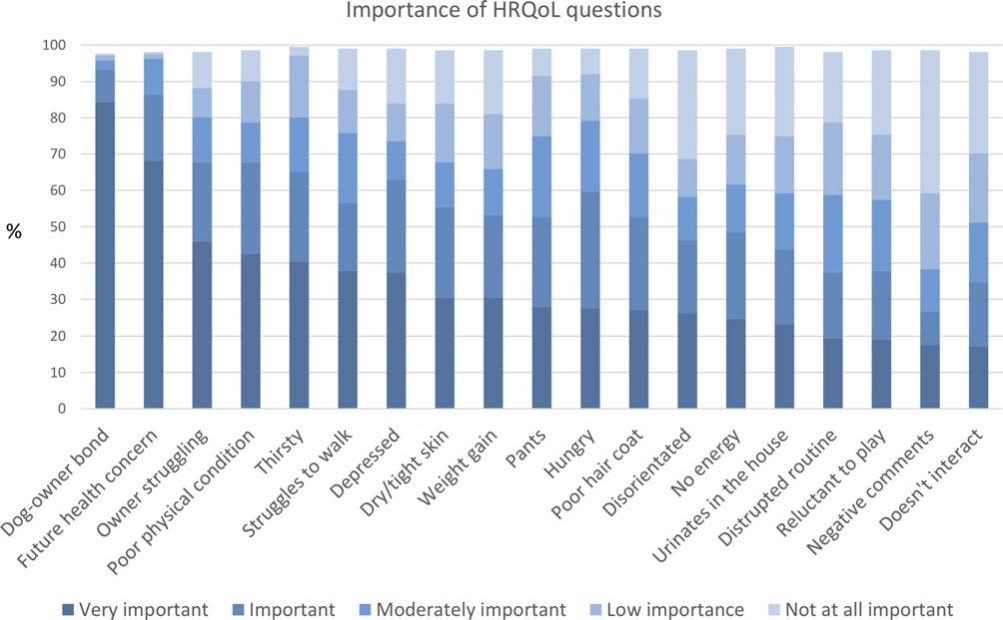
Proportional responses of perceived HRQoL question importance to owners and their dogs with Cushing's syndrome (n = 210)

The questions reported by owners of dogs with Cushing's syndrome as most important were those that explored whether Cushing's syndrome affects the bond with their pet and how much they worry about their pet's future health (Figure 3). The least important items were about their pet's appearance and interaction with other people/dogs. When assessing the reliability of paired owner reported HRQoL question importance, there was moderate to poor agreement of inter‐rater (ICC = .53, 95% CI −.77 to .88) and intra‐rater assessments (ICC = .54, 95% CI −.55 to .91). Increasing age, having a comorbidity, or increasing length of time as diagnosis were not statistically associated with having a better HRQoL in dogs with Cushing's syndrome (*P* = .84, .34, and .08, respectively). Dogs currently on treatment with trilostane (.33, IQR .23–.44) were reported to have a better HRQoL than those on alternative medical treatment or no treatment (.36, IQR .33–.54, *P* = .04) (Table 4).

**Table 4:** Factors associated with HRQoL score in dogs with Cushing's syndrome (n = 210)

Variable	Cases (%)	Median HRQoL score (IQR)	*P* value^1^
Trilostane			.04
Yes	159 (77.2)	.33 (.23–.44)	
No	47 (22.8)	.36 (.33–.54)	
Age			.84
<7	17 (8.2)	.37 (.28–.53)	
7 to ≤11	103 (50.0)	.37 (.23–.47)	
>11	86 (41.8)	.35 (.26–.44)	
Comorbidity			.34
Yes	138 (66.7)	.35 (.25–.47)	
No	69 (33.3)	.33 (.25–.42)	
Time since diagnosis			.08
≤1 month	23 (11.2)	.39 (.33–.58)	
>1–12 months	80 (38.8)	.35 (.23–.49)	
>12 months	103 (50.0)	.33 (.25–.44)	

1 Nonparametric test *P* value.

## 4 DISCUSSION

The developed CushQoL‐pet quantifies the HRQoL of dogs with Cushing's syndrome and can be a useful tool for clinicians and researchers to aid clinical assessment of dogs with Cushing's syndrome. The final 19‐question tool was shown to be interpretable, valid, and reliable for owner‐completion in a population of dogs with Cushing's syndrome. These are deemed important qualities of a quality‐of‐life tool^8^, ^12^, ^29^; however, they are infrequently assessed in published quality‐of‐life assessments in dogs.^29^ The internal consistency of CushQoL‐pet indicated good reliability to measure the same latent concept; “HRQoL of dogs with Cushing's syndrome” (α = .83). Inter‐rater, intra‐rater, and test‐retest assessments of the tool further indicated reliability of owner completion of CushQoL‐pet. The overall reliability of the inter‐rater was slightly higher than the intra‐rater reliability. This suggests that 2 different owners had better agreement than the same owner repeating the questionnaire twice within a 2 week time period. However, the reverse could have been expected. This could be that changes in the dogs' HRQoL were truly observed in a 2 week time period. Another suggestion could be that owners repeating the questionnaire over a short time period changed their behavior when familiarized with the questions and became accustomed to the format. The inter‐rater scores appeared to have greater agreement for higher scores, indicating a poorer HRQoL, than lower scores when examining the Bland‐Altman plot. This suggests that 2 owners had the greatest agreement on their pet's HRQoL when it was poor. These reliability assessments would be interesting to explore further with a larger sample size.

The CushQoL‐pet score of dogs with Cushing's syndrome showed a general increasing trend with poorer owner‐perceived quality‐of‐life, further validating the tool. However, the moderate correlation (*r* = .41) could suggest the value of more detailed assessment encompassing the multiple facets of HRQoL, above a singular direct question about overall quality‐of‐life. There was also a difference observed in the CushQoL‐pet score between dogs with and without Cushing's syndrome (*P* < .001). When examining dogs without Cushing's syndrome by different age groups and health status, there was still a significant difference in their CushQoL‐pet score. However, there was no difference in CushQoL‐pet scores of those with Cushing's syndrome across different age groups or with comorbidities (*P* = .84 and .34, respectively). This indicates that the HRQoL described by CushQoL‐pet is specific to Cushing's syndrome and suggests the score is not highly influenced by the dog's age or other morbidities which has been a concern regarding the application of disease‐specific HRQoL tools.^29^ When comparing the changes in the median test‐retest scores with the owner assessment of the change in their dog's quality‐of‐life over the same time period, the tool was able to detect the direction of change (either improvement or deterioration). The median score differences suggested that the tool was better at indicating improvement in HRQoL than a deterioration in HRQoL. A decreased score of about −.10 indicated owner‐assessed improvement and an increase of +.05 indicated deterioration. This study focused purely on owner‐reported HRQoL, unaffected by veterinarian's opinions, as this type of reporting is currently lacking in the veterinary literature. Nevertheless, the lack of veterinarian assessment of health status is a potential limitation of this study. Future replication of results within a practice setting, alongside veterinarian evaluation of a clinical assessment, could provide further evidence of reliability and validity.^30^ In particular, assessment of changes in owner questionnaire response behavior over time and evaluation of CushQoL‐pet's responsiveness to changes in HRQoL would be of interest.^31^

The design of the tool was intended for it to be quick for owners to complete, as well as being easy to interpret for veterinarians to encourage its uptake in primary‐care practice. The tool is comparable in length to other HRQoL tools,^13^, ^14^, ^32^ with some other published quality‐of‐life tools noticeably longer.^11^, ^33^, ^34^ The average time to complete the questionnaire during this study was 6 minutes. However, this included a number of additional questions that will not be included in the final version used in practice and therefore completion of the CushQoL‐pet in a clinical setting is likely much shorter than this. A suggested integration of the tool into practice would be during therapeutic monitoring consultations. During refinement of the tool, 7 questions were removed as they were either shown to be poorly correlated with the other questions, not specific to the Cushing's syndrome dog population or were highly correlated with another question, indicating repetition. A recent study found that the shortening of a much longer tool was valid and would likely increase its acceptability.^35^ A scoring system on a 0–1 scale was used without weighting of the questions to ensure the final score was easy to calculate and interpret within primary‐care practice.^8^, ^36^

Three principal components were retained for further analysis with 3 subsets of questions identified within the tool. Although the individual factor loadings identified were weakly correlated to the overall component (.30 ≤ *r* < .45), the subsets were clinically justifiable and generally had good internal consistency. The subset structures and the reliability estimates provide evidence of internal coherence and construct validity of the tool. Questions related to the demeanor of dogs with Cushing's syndrome described the largest explanatory principal component in the PCA. This could indicate that demeanor should be given increased emphasis in clinical evaluation of affected dogs and highlights an interesting parallel with the human situation where depression is thought to have a substantial impact on HRQoL, with the resulting hypercortisolism associated with psychiatric and neurocognitive disorders in human patients with Cushing's syndrome.^37^ Fatigability and muscle weakness have a detrimental effect on the HRQoL of people with Cushing's syndrome.^17^, ^38^ With some similarities between Cushing's syndrome in people and dogs, there is the possibility that these more subtle physiological and psychological effects could be overlooked in dogs. Therefore, a comprehensive assessment of the HRQoL in dogs with Cushing's syndrome could bring certain properties of the disease to light.

Areas described of highest importance to owners and their pets generally related to areas of “owner impact.” This is interesting but perhaps unsurprising, reflecting similar findings in other studies.^10^, ^39^ Owner‐related questions included within the HRQoL tool were those that impact upon the dog and potentially affect how Cushing's syndrome is managed.^40^ Owner factors deemed relevant were determined in the pretesting of the questionnaire with expert opinions across a range of disciplines. These include the current bond between the owner and dog and how well owners feel they are managing their dog's health. In veterinary medicine, direct evaluation of quality‐of‐life is not possible (dogs cannot directly communicate how they feel), therefore individualization of a HRQoL tool for animals is difficult. It was decided not to weigh the tool by incorporating owner assessed importance into the final tool which could have taken individualized dog needs and preferences into account.^29^ Importance score reliability assessments indicated these questions were subjective, with disagreement between 2 owners' views (ICC = .53, 95% CI −.77 to .88) and variations in repeated owner response within a 2‐week period (ICC = .54, 95% CI −.55 to .91) therefore affecting the overall score reliability. Additionally the increased perceived importance of owner specific factors potentially highlighted a limitation of proxy reporting by owners. It cannot be assumed that proxy assessment will be a true reflection of the HRQoL of a dog with Cushing's syndrome. Studies evaluating quality‐of‐life in children via proxy have compared the results to the individuals own experience with varying views of the assessment.^41^, ^42^ The owner rather than the veterinarian was used as the proxy in the current study because of their closer relationship and time spent with their pet.

Current age, having a comorbidity and time since diagnosis, was not associated with the HRQoL of dogs with Cushing's syndrome. There are no studies currently that have quantitatively examined risk factors associated with a poorer HRQoL in dogs with Cushing's syndrome. A recent study examined the factors affecting euthanasia decisions in dogs with diabetes mellitus finding age, concurrent disease, and costs were considered of high importance by clinicians.^43^ It could be assumed that similar factors would have been associated with the HRQoL of dogs with Cushing's syndrome. Additionally, it could be suspected that age would have a negative effect on HRQoL because of its association with reduced survival.^44^, ^45^, ^46^ Treatment with trilostane was weakly associated with a better HRQoL (*P* = .04) and the difference detected only small (.03). Studies examining survival and clinical responses to trilostane are favorable; therefore, this could truly be a reflection of improved HRQoL in trilostane treated dogs.^46^, ^47^, ^48^, ^49^ The duration of time dogs have been on treatment and the reasons for dogs in this population not receiving trilostane are unknown; therefore, the weak association found might be confounded when taking other factors into consideration. Future examination of changes in HRQoL before and after commencement of trilostane in a clinical setting would be interesting.

The study had some limitations. As with any questionnaire, there is the potential for recall bias.^50^, ^51^ This was considered in the study methodology by not mentioning planned assessment of quality‐of‐life in the introduction to avoid owners answering questions with a preconceived view of quality‐of‐life. The methods of recruitment used could have resulted in selection bias of the owners that participated. Owners participating in this study might not have been representative of the general underlying population. The proportion of incomplete responses was greater for owners of dogs without Cushing's syndrome. This could be because owners of dogs with Cushing's syndrome would be more invested in this research than owners of control dogs. Awareness of the questionnaire was raised through a variety of different sources and recruitment did include some social media and webpage promotion. The methods of promotion via this method were directed as much as possible, primarily using veterinary sites to target veterinary professionals and owners of dogs with Cushing's syndrome (such as the Veterinary Information Network, Dechra Veterinary Products Ltd, and the Royal Veterinary College). The use of these platforms was to reach a large numbers of owners of dogs with Cushing's syndrome, which is not a highly prevalent disease within veterinary practices.^52^ Targeted focus on veterinary practice promotion of the study to clients aimed to increase the likelihood of a veterinarian confirmed diagnosis of Cushing's syndrome as well as to increase the representativeness of participation. However, it is possible that some owners might have completed the tool without their dog having received a veterinary‐confirmed diagnosis of Cushing's syndrome. Selection bias could have resulted because responses were only completed online. The majority of respondents were from the United Kingdom and the second largest proportion was from the United States. Inclusion of respondents across several countries was deemed to provide a broad view of the HRQoL of Cushing's syndrome.

## 5 CONCLUSIONS

In conclusion, CushQoL‐pet is the first tool to quantify the HRQoL of dogs with Cushing's syndrome. The validated tool can be used within practice and research to aid clinical assessment of dogs with the disease and could provide a supplementary tool to current monitoring methods.

## CONFLICT OF INTEREST DECLARATION

Imogen Schofield is supported at the Royal Veterinary College by an award from Dechra Veterinary Products Ltd. Dechra Veterinary Products Ltd did not have any input in the design of the study, analysis and interpretation of data or in writing the manuscript. Dechra Veterinary Products Ltd did aid in the promotion of the questionnaire.

## OFF‐LABEL ANTIMICROBIAL DECLARATION

Authors declare no off‐label use of antimicrobials.

## INSTITUTIONAL ANIMAL CARE AND USE COMMITTEE (IACUC) OR OTHER APPROVAL DECLARATION

Approval was granted by the Royal Veterinary College Ethics and Welfare Committee (URN 2015 1373).

## HUMAN ETHICS APPROVAL DECLARATION

Authors declare human ethics approval was not needed for this study.

## Supporting information


**Appendix S1.** Supporting Information.
