# Supplementary material for: Development and evaluation of a health‐related quality‐of‐life tool for dogs with Cushing's syndrome
Source: J Vet Intern Med. 2019 Oct 29;33(6):2595–604. doi: 10.1111/jvim.15639 (PMC6872869; doi:10.1111/jvim.15639)
Supplement: Supplementary file 1 — Appendix S1. Supporting Information. [file JVIM-33-2595-s001.pdf]

**Initial items**	**Retained**	**Excluded**	**Reason for exclusion**
My dog is excessively thirsty	x		
My dog drinks his/her water bowl dry:		x	Highly correlated with 'my dog is excessively thirsty' (r = .68)
My dog urinates excessively:		x	Poor inter-item correlations (r = .29)
My dog urinates in the house:	x		
My dog begs for food and scavenges:		x	Poor inter-item correlations (r = .22)
My dog is excessively hungry:	x		
My dog pants excessively:	x		
My dog appears to be gaining weight:	x		
I struggle to control my dogs weight		x	Removed in pilot stage. Repetitive of 'my dog appears to be gaining weight' but less specific regarding weight gain or loss.
My dog is depressed and quiet:	x		
My dog has no energy:	x		
My dog's behavior has changed:		x	Remove in pilot stage. Other questions relate to this and non-specific to how behaviour has changed.
My dog doesn't want to interact with people/other dogs:	x		
My dog is reluctant to play with me:	x		
My dog seems disorientated/confused:	x		
My dog's hair coat is in a poor condition:	x		
My dog's skin appears to be uncomfortable (e.g. dry/ tight/ itchy):	x		
My dog is in poor physical condition (e.g. muscle loss/big belly):	x		
I feel my dog's appearance gets negative comments:	x		
My dog struggles to walk very far:	x		
My dog is weak (e.g. cannot jump into the car/on the sofa or cannot get upstairs):		x	Highly correlated with 'my dog struggles to walk very far' (r = .63)
My dog has more sickness or diarrhoea than is normal for him/her:		x	Removed in pilot stage. Not specific to Cushing's syndrome.
My dog is disinterested in his/her food:		x	No difference in dogs with or without Cushing's (p = .33)
My dog gets stressed or reacts to having medication/tablets:		x	No difference in dogs with or without Cushing's (p = .22)
The appointments at the vets are stressful for my dog:		x	Poor inter-item correlations (r = .23)
Because of the care I give to my		x	Removed in pilot stage. Not deemed necessary.

dog, my social/working life are affected:			
I worry about the future health of my dog:	x		
Mine and my dog's daily routine is currently being disrupted:	x		
Currently I choose not to take my dog out with me as much as I used to:		x	Removed in pilot stage. Not deemed necessary.
I feel like I do not have control around the treatment decisions regarding my dog:		x	Removed in pilot stage. Deemed to be a leading question.
I feel I am struggling to manage my dog's health:	x		
Currently I feel there is a strong bond between me and my dog:	x		

All 32 items initially identified for inclusion in the online questionnaire which were subsequently retained or excluded from the final tool, CushQoL-pet.
